# Fontan-Kreutzer Conversion to Total Cavopulmonary Surgery due to Failing Univentricular Circulation. A Feasible Therapeutic Option?

**DOI:** 10.5935/abc.20190021

**Published:** 2019-02

**Authors:** Isabel Cristina Britto Guimarães

**Affiliations:** 1 Universidade Federal da Bahia - Faculdade de Medicina da Bahia, Salvador, BA - Brazil; 2 Hospital Ana Nery - Universidade Federal da Bahia, Salvador, BA - Brazil

**Keywords:** Heart Defects, Congenital, Tricuspide Atresia/surgery, Procedure Fontan-Kreutzer, Heart Bypass, Right

Fontan surgery, a palliative operation for patients with “single-ventricle” heart physiology, has undergone several modifications since the first procedure was performed in 1971 for individuals with diagnosis of tricuspid atresia.^1^

The Fontan-Kreutzer (FK) atrial-pulmonary anastomosis technique was widely used in the 1980s. Publications regarding the late follow-up of patients submitted to the technique before 1990 showed a higher frequency of complications such as heart failure, arrhythmias, thromboembolic events, protein-losing enteropathy, plastic bronchitis, sudden death and liver failure.^2,3^

The study by Fernandes et al.,^4^ published in this issue of the journal, aimed to analyze the results of the conversion of FK to Total Cavopulmonary Connection (TCPC) of patients with signs of failing univentricular circulation submitted to surgery in a single institution.

Of the 420 patients submitted to Fontan surgery between 1995 and 2016, 18 underwent FK, corresponding to 4.3% of the total sample. Of the 18 FK cases, 10 required conversion to TCPC due to signs of failing Fontan circulation, all of which were included in the study analysis. In 9 cases, the main cause of conversion was the presence of uncontrolled arrhythmia and protein-losing enteropathy in 1 case.

A relevant aspect regarding the presented data concerns the New York Heart Association functional class. Before the surgical conversion, 70% of the patients were in functional class II and III, and after the conversion surgery, approximately 80% of the patients showed functional capacity improvement.

Although the main indication for conversion was the presence of difficult-to-control arrhythmias, about 44% of the patients continued to have arrhythmias as a clinical manifestation, showing no satisfactory results with the use of surgical cryoablation, different from studies showing favorable results after its use.^5^ The obtained results also show data that demonstrate the complexity of these patients’ management, such as: prolonged hospitalization time and death rate of 20%. The authors associated the mortality rate found in the study with the period during which the surgeries were performed between 1996 and 2000, and the learning curve of the service related to the described surgical technique. However, the early mortality rate described by other authors was also high, ranging from 0 to 21%.^6,7^

Kreutzer et al.,^8^ in a review article, assess the five decades of the FK technique, in which they state that late complications would be strongly associated with surgical strategies and procedures no longer used nowadays, such as prolonged use of pulmonary artery banding, classic Blalock-Taussig shunt, late interventions, late diagnosis of significant hemodynamic changes and the use of surgical techniques currently considered to be inadequate, such as the classic Fontan procedure and the original Kreutzer surgery. Kreuzter et al.^8^ consider that one should be careful when analyzing literature data regarding the surgical technique prior to 1990, as well as when using these results as predictors of long-term outcome in patients submitted to FK surgery today.^8^

Regarding the study by Fernandes et al.,^4^ we do not have this information, which could help us to better understand the late complications observed in this sample.

For the late survivors of the "old-fashioned" FK technique, Kreutzer et al.^8^ consider that the conversion to TCPC would be indicated in cases with arrhythmia, symptomatic ones, and those unresponsive to treatment with amiodarone and in the presence of thrombus in the right atrium.^8^ Heart transplant after Fontan has been considered a therapeutic option in cases of which the main determinant of failure is ventricular dysfunction and some centers have already shown favorable results.^9,10^ In the study by Fernandes et al.,^4^ one patient is awaiting a cardiac transplant after a pacemaker was implanted, with an unfavorable evolution.

Fontan conversion strategy has been described since 1991.^9^ Worldwide, there is limited experience with this procedure, largely restricted to a small number of centers, and even in services with a greater number of surgical procedures, they have usually followed only a few dozen patients.^6^ The long-term evolution and the best time to perform the conversion is still a matter of debate.^8,9^ In a retrospective analysis of ten years of follow-up of patients undergoing Fontan conversion, using Australian and New Zealand registries, Poh CL et al.,^5^ demonstrated that patients submitted to an earlier conversion had more favorable long-term outcomes, with a heart transplant-free 10-year survival of 86%.^5^

A systematic review carried out by Brida et al.,^7^ which analyzed 1,182 patients, concluded that Fontan conversion has a high mortality risk and the combination with arrhythmia surgery seems to be associated with lower early mortality, especially when patients are referred at an early age and treated in centers of expertise.^7^

Regardless of the sample size analyzed in the article, the results are comparable to the literature data, demonstrating the complexity of the procedure and the importance of its performance in more experienced centers.^11^
